# Epigenetic Regulation of ATP-Binding Cassette Protein A1 (*ABCA1*) Gene Expression: A New Era to Alleviate Atherosclerotic Cardiovascular Disease

**DOI:** 10.3390/diseases6020034

**Published:** 2018-05-03

**Authors:** Mohamed Zaiou, Ahmed Bakillah

**Affiliations:** 1 School of Pharmacy, University of Lorraine, 5 rue Albert Lebrun, 54000 Nancy, France; 2 Department of Medicine, Downstate Medical Center, State University of New York, 450 Clarkson Ave, Brooklyn, NY 11203, USA; Ahmed.Bakillah@downstate.edu

**Keywords:** ABCA1, HDL, miRNA, circular RNA, gene expression, RNA-binding proteins, reverse cholesterol transport, cardiovascular diseases

## Abstract

The most important function of high density lipoprotein (HDL) is its ability to remove cholesterol from cells and tissues involved in the early stages of atherosclerosis back to the liver for excretion. The ATP-binding cassette transporters ABCA1 and ABCG1 are responsible for the major part of cholesterol efflux to HDL in macrophage foam cells. Thus, promoting the process of reverse cholesterol transport (RCT) by upregulating mainly ABCA1 remains one of the potential targets for the development of new therapeutic agents against atherosclerosis. Growing evidence suggests that posttranscriptional regulation of HDL biogenesis as well as modulation of ABCA1 expression are under the control of several genetic and epigenetic factors such as transcription factor (TFs), microRNAs (miRNAs) and RNA-binding proteins (RBPs).These factors may act either individually or in combination to orchestrate ABCA1 expression. Complementary to our recent work, we propose an exploratory model for the potential molecular mechanism(s) underlying epigenetic signature of ABCA1 gene regulation. Such a model may hopefully provide the basic framework for understanding the epigenetic regulation of RCT and contribute to the development of novel therapeutic strategies to alleviate the burden of cardiovascular diseases (CVD).

## 1. Introduction

Based on the general consensus that HDL protects against atherosclerotic CVD, several attempts have been made to design drugs that raise HDL cholesterol (HDL-C) levels or enhance its cardioprotective function. However, several prospective HDL-C-raising trials have failed to demonstrate improved efficacy for major adverse cardiovascular outcomes. In this prospect, there are continuous efforts to increase HDL functionality to enhance RCT and potentially achieve further cardiovascular event reduction.

Over the last decades, genetic breakthroughs have revolutionized cardiovascular research by providing opportunities to elucidate novel molecular mechanisms underlying HDL biogenesis and RCT pathways. Some preclinical and clinical studies have shown that apoA-I Milano, a naturally occurring mutant of ApoA-I (Arg173Cysteine) has beneficial athero-protective and anti-inflammatory effects [1]. Furthermore, research has suggested that the expression of genes involved in the RCT combines several complex regulatory networks that are controlled by at least two types of trans-factors: transcription factors (TFs) in the nucleus and posttranscriptional epigenetic factors that bind to cis-regulatory RNA elements mostly located in the 3′UTR of their target mRNAs. Epigenetic factors including miRNAs and RBPs have been shown to be associated with several pathophysiological conditions. For instance, the dysregulation of miRNAs has been associated with the disruption of multiple gene networks leading to metabolic disorders including diabetes, obesity, metabolic syndrome and atherosclerosis [2,3]. This clearly indicates that beyond the existing therapies, advance in epigenetics mechanisms could offer additional opportunities to develop novel treatment strategies for atherosclerosis. In this regard, attempts have been made to develop Apabetalone (RVX-208) as the first epigenetic approach to treat CVD. In recent clinical trials such as SUSTAIN and ASSURE, RVX-208, an orally active molecule has been shown to increase plasma apoA-I, HDL-C (pre-beta and alpha-HDL), and enhance the ability of serum to efflux cholesterol via ABCA1, ABCG1, and scavenger receptor class B type I (SR-BI)-dependent pathways [4]. RVX-208 increases apoA-I transcription through an epigenetic mechanism by inhibiting the bromodomain and extra-terminal domain (BET) protein 4 (BRD4) [5]. Most importantly, emerging evidence has suggested that miRNAs act as a novel class of epigenetic regulators of RCT and HDL-C from synthesis to clearance, and thus contributing remarkably to the pathogenesis of atherosclerosis [6]. Hence, understanding epigenetic mechanisms that control RCT genes network, mainly *ABCA1* gene that is involved in the initiation of this process, may bring insights into novel therapeutic approaches for treating atherosclerotic vascular disease.

## 2. Discussion

**Figure 1:**
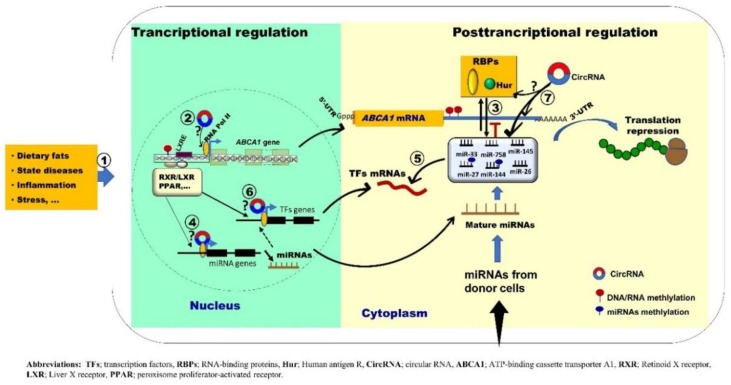
Schematic representation of proposed scenarios for the mechanisms by which *ABCA1* gene expression is regulated. Various factors including nutrition, state diseases, stress and inflammation can alter epigenetic *ABCA1* gene regulation in different cells mainly macrophages and hepatocytes (**1**). At the transcriptional level, *ABCA1* gene expression is regulated by key nuclear receptors including LXR family and their heterodimeric partners, retinoic acid receptors (RXR) via functional LXREs (**2**). At the post-transcriptional level, miRNAs and RBPs (Hur) cooperate/compete for target binding regulation (**3**). miRNAs and TFs may cooperate to tune gene expression by forming feedback or feedforward loops (**4**) and (**5**). CircRNAs may also be part of the interplay by their potential interaction with TFs, miRNAs and RBPs (**2**), (**4**), (**6**) and (**7**). Finally, the proposed interplay between TFs-miRNAs-RBPs-CircRNAs could be taken into consideration while elucidating epigenetic mechanisms regulating the first step of RCT gene network.

ABCA1 is a key mediator of cholesterol efflux to lipid-poor apolipoprotein A-1. Several studies have confirmed that compromised ABCA1 activity leads to accelerated and early atherogenesis. As summarized in our recent paper [6], the RCT gene network is a complex process highly controlled at both transcriptional and posttranscriptional levels. Herein, we consider the *ABCA1* gene, for which some experimental knowledge is available both at the transcriptional and posttranscriptional levels, as an example to illustrate potential epigenetic mechanisms driving the regulation of RCT. We propose a hypothetical model for the potential dynamic interplay between different genetic and epigenetic regulators that may serve to regulate *ABCA1* gene enabling the cell to respond to different environment changes (Figure 1). However, all proposed axes of interaction, if they occur, need to be experimentally validated in different models.

At the transcriptional level, several nuclear receptors including peroxisome proliferator-activated receptors (PPARs), liver X-receptor (LXR), and farnesoid X receptor (FXR), have been shown to influence lipid metabolism along with genes involved in RCT pathway including *ABCA1*. Unfortunately, despite the effectiveness of these nuclear receptors in preclinical studies, their translation to human clinical trials is still facing many challenges.

At the posttranscriptional level, the 3′-UTR of *ABCA1* gene has been shown to be directly targeted by multiple miRNAs including miR-33, miR-758, miR-145, miR-27, miR-144, miR-26 and miR-106, which lead to cholesterol efflux and HDL-C levels repression [6]. In addition to miRNAs, RNA-binding proteins (RBPs) are also known to bind to AU-rich elements (AREs) in the 3′UTR of many genes, and thereby modulate their expression by increasing or decreasing mRNAs’ translation and/or stability. With respect to ABCA1, human antigen R (HuR), an RBP, has been reported to bind to the 3′-UTR of this transporter and increase its expression by enhancing protein translation [7]. Based on this information, it would be tempting to suggest the existence of a possible regulatory relationship between miRNAs and RBPs that could influence ABCA1 expression. In this context, specific miRNAs and RBPs may act via cooperation/competition to directly or indirectly regulate gene expression. Furthermore, post-translational modifications of RBPs, including their phosphorylation and methylation, provide additional layers of complexity, as they control RNA-binding, function and localization [8]. Therefore, phosphorylation and nuclear transit of RBPs could be another possible mechanism to influence RBP-mediated regulation of *ABCA1* gene expression as suggested in a previous study [9]. In addition to these potential mechanisms, certain miRNA species may also control the expression of other important epigenetic regulators such as DNA methyltransferases and histone deacetylases. Conversely, DNA/RNA methylation and histone modification may contribute to the regulation of these miRNAs.

As is true for protein-coding genes, the expression of miRNAs is also under the control of numerous transcription factors. Aberrant regulation of miRNAs by TFs can cause phenotypic variations and diseases. As an example, miR-26, known to directly target the *ABCA1* 3′-UTR, thereby repressing cholesterol efflux and HDL-C levels, has been shown to be inhibited in cells treated with LXR agonists [10]. Moreover, the activation of LXR has been associated with an increase in miR-144 and ABCA1 expression to fine-tune RCT by macrophages [11]. On the other hand, miRNAs/TFs feedback-loop regulation may occur and represent another possible mechanism involved in the regulation of the RCT gene networks [12]. As an example of the TFs–miRNA regulatory network, RXRα has been reported to be regulated by miRNAs including miR-128-2 [13], while other miRNAs have been shown to regulate the expression and activity of different TFs such PPARs. Hence, it is reasonable to assume the existence of a possible cross-talk between TFs and miRNAs that could modulate *ABCA1* gene and RCT circuit. However, such mechanisms of regulation are still not well defined and could represent an important future area of research.

Circular RNAs (circRNAs), a class of RNAs with the linking of 3′ and 5′ ends, are predicted to function as robust transcriptional and posttranscriptional regulators of gene expression. Evidence is arising that some circRNAs might regulate miRNA function as microRNA sponges. Relevant information in this respect can be provided by the web tool CircInteractome, freely available at http://circinteractome.nia.nih.gov. This new database has been developed for exploring circRNAs and their interacting proteins and microRNAs [14]. Further studies have suggested that circRNA-miRNA-mRNA axes play a prominent role in different pathologies including CVD [15]. Some circRNAs have been predominantly localized in the nucleus where they can directly promote host-gene transcription through interaction with RNA polymerase II (pol II) in the promoter region of genes. Furthermore, these RNAs may interact with TFs to influence various diseases [16].

Because no data are yet available regarding the impact of circRNAs on lipid homeostasis and HDL biogenesis, this has prompted us to propose a model integrating TFs, miRNAs, RBPs and CirRNAs into the *ABCA1* regulatory gene network (Figure 1). However, we are aware that certain circuits of this model are much more difficult to directly translate in vivo mostly due to the complexity of several highly connected pathways with feedback and feedforward loops. Thus, to validate the proposed epigenetic transcriptional and post-transcriptional mechanisms for *ABCA1* gene expression regulation, future well-designed in vivo experimental studies combined with high-throughput sequencing and bioinformatic data are required.

## 3. Conclusions and Perspectives

Emerging research suggests that deregulated miRNAs can impact RCT gene networks, mainly *ABCA1* gene. These observations have generated a renewed interest in novel targets for epigenetic regulators that may pave the way for establishing novel strategies to raise functional HDL and promote RCT. However, since the field of epigenetic regulation by small RNAs is still in its infancy, there are only a few experimentally validated data associating the dysregulation of miRNAs with genes involved in the HDL biogenesis and RCT. Our proposed model (Figure 1) for potential epigenetic regulation of *ABCA1* is expected to be translated into specific questions and hypothesis which may benefit further research in this area. However, one of the greatest challenges one may confront here is how to fully gain knowledge from the potential interplay and/or cross-talk between various miRNAs and other cellular regulatory factors controlling specific pathway(s) of cholesterol removal from cells and how all these networks can shape a physiological function in both normal and pathological cells in response to a behavior or a stimulus. To answer these questions, more focus should be given to the integration of computational approaches such as systems biology and molecular networks modeling in combination with high-throughput experiments to provide a list of potential miRNA target genes involved in the RCT process. This might help us to better understand the consequences of the complex interplay of miRNAs with other epigenetic regulators on *ABCA1* gene and possibly other relevant genes involved in RCT regulation.
